# Supplementary material for: Physical Performance and Skeletal Muscle Transcriptional Adaptations Are Not Impacted by Exercise Training Frequency in Mice with Lower Extremity Peripheral Artery Disease
Source: Metabolites. 2023 Apr 16;13(4):562. doi: 10.3390/metabo13040562 (PMC10143072; doi:10.3390/metabo13040562)
Supplement: Supplementary file 1 [file metabolites-13-00562-s001.zip › metabolites-2290913-supplementary.pdf]

metabolites

MDPI

Article

Physical Performance and Skeletal Muscle Transcriptional Adaptations Are Not Impacted by Exercise Training Frequency in Mice with Lower Extremity Peripheral Artery Disease

Jessica Lavier, Karima Bouzourène, Grégoire P Millet, Lucia Mazzolai and Maxime Pellegrin

Table S1. Mouse primer sequences used for quantitative real-time PCR.

Gene	Forward sequence	Reverse sequence
36B4	5′-ATGGGTACAAGCGCGTCCTG-3′	5′GCCTTGACCTTTTCAGTAAG-3′
GYS1	5′-GAGAACGCAGTGCTTTTCGA-3′	5′-TCATCCCCTGTCACCTTCG-3′
GLUT-1	5′-GTGTATCCTGTTGCCTTC-3′	5′-GCTTCTTCAGCACACTCTT-3′
GLUT-4	5′-CTCTCAGGCATCAATGCTGTTTTCTA-3′	5′-CGAGACCAACGTGAAGACCGTATT-3′
HK2	5′-GCTAGGAGCTACCACACACCCT-3′	5′-ACTCGCCATGTTCTGTCCCATCC-3′
PFK	5′-GGAGTGCGTGCAGGTGACCAAA-3′	5′-ATCACGGCCACTGTGTGCAACC-3′
PDK4	5′-TACTCCACTGCTCCAACACCTG-3′	5′-AGCCATAACCAAAACCAGCCAAAG-3′
LDHA	5′-TGCCTACGAGGTGATCAAGCT-3′	5′-GCACCCGCCTAAGGTTCTTC-3′
LDHB	5′-AGTCTCCCGTGCATCCTCAA-3′	5′-AGGGTGTCCGCACTCTTCCT-3′
MCT1	5′-TTGTCTGTCTGGTTGCGGCTT-GATCG-3′	5′-GCCCAAGACCTCCAATAACAC-CAATGC-3′
MCT4	5′-GCCACCTCAACGCCTGCTA-3′	5′-TGTCGGGTACACCCATATCCTTA-3′
CD36	5′-ATGGGCTGTGATCGGAACTG-3′	5′-GTCTTCCCAATAAGCATGTCTCC-3′
FABP3	5′-ACCTGGAAGCTAGTGGACAG -3′	5′-TGATGGTAGTAGGCTTGGTCAT-3′
CPT1β	5′-TGGACCGTGAAGAGATCAAGC-3′	5′-CTCTTTGCCTGGGATGCGT-3′
HSL	5′-ACTGAGATTGAGGTGCTGTC-3′	5′-TGAGATGGTAACTGTGAGCC-3′
UCP2	5′-TTCCCTGTTGATGTGGTCAA-3′	5′-CAGTGACCTGCGCTGTGGTA-3′
LCAD	5′-CATATTCCCCCAGGACATTG-3′	5′-CACAATTGCCTCTATGTGCATT-3′
PPAR-γ	5′-ACCACTCGCATTCCTTTGAC-3′	5′-TGGGTCAGCTCTTGTGAATG-3′
PGC-1α	5′-ACTATGAATCAAGCCACTACAGAC-3′	5′-TTCATCCCTCTTGAGCCTTTCG-3′
PGC-1β	5′-GAGGAGTCCCTTCCTTCATC-3′	5′-TCCTCGAAGGTTAAGGCTGA-3′
NRF1	5′-GCACCTTTGGAGAATGTGGT-3′	5′-CTGAGCCTGGGTCATTTTGT-3′
TFAM	5′-CCAAAAAGACCTCGTTCAGC-3′	5′-CTTCAGCCATCTGCTCTTCC-3′
CS	5′-GGACAATTTTCCAACCAATCTGC-3′	5′-TCGGTTCATTCCCCTCTGCATA-3′
MyH7	5′-CCTGCTGTTTCCTTACTTG-3′	5′-CTGTACTCCTCTGCTGAG-3′
MyH2	5′-CTGACTCGTCCTGCTTTA-3′	5′-CTCACAGACCCTTACTGG-3′
MyH1	5′-AAAGCTTCAAGTTTGGACC-3′	5′-GAGAGAGCCTGCCTTTAG-3′
MyH4	5′-GTCCTTCCTCAAACCCTTA-3′	5′-CTCTTGTGTTACCAGATGAAG-3′
VEGFA	5′-TGCACCCACGACAGAAGG-3′	5′-GCACACAGGACGGCTTGA-3′
HIF-1α	5′-TCAAGTCAGCAACGTGGAAG -3′	5′-TATCGAGGCTGTGTCGACTG-3′
IL-1β	5′-TGAAGTTGACGGACCCCAAAA-3′	5′-TGATGTGCTGCTGTGAGATT-3′
CD11c	5′-ACACAGTGTGCTCCAGTATGA-3′	5′-GCCCAGGGATATGTTCACAGC-3′
IL-10	5′-GCACTACCAAAGCCACAAGGC-3′	5′-GTCAGTAAGAGCAGGCAGCATAG-3′
IL-1ra	5′-ACAGTAGAAGGAGACAGAAG-3′	5′-CCTGGTAGAGCAGAAGAC-3′
